# *CD34* is not Expressed by Blasts in a Third of B-ALL Patients and Its Negativity is associated with Aberrant Marker Expression: A Retrospective Analysis

**DOI:** 10.31557/APJCP.2021.22.3.919

**Published:** 2021-03

**Authors:** Neha Garg, Richa Gupta, Mrinalini Kotru

**Affiliations:** *Department of Pathology, University College of Medical Sciences & Guru Teg Bahadur Hospital, Dilshad Garden, New Delhi, India. *

**Keywords:** CD34- B, acute lymphoblastic leukemia, acute leukeimia, immunophenotype, flow cytometry, aberrant

## Abstract

**Background:**

*CD34* antigen is expressed by early hematopoietic progenitor cells and acute leukemia cells. Its expression is associated with good prognosis in acute myeloid leukemia. Literature is sparse on its prognostic significance in B- acute lymphoblastic leukemia (B-ALL) especially from India. Hence the present study was undertaken to analyse the frequency of *CD34* expression in B-ALL in Indian patients and determine its prognostic significance by associating with other prognostic markers and aberrant antigen expression.

**Methods:**

Seventy-five B-ALL patients diagnosed by flow cytometry over a period of 3½ year were studied. Correlation of *CD34* expression was studied with gender, age, total leucocyte count (TLC), French-American-British (FAB) morphological type, immuno-phenotypic markers, cytogenetics and minimal residual disease. Differences between groups were evaluated using Student’s T-test for quantitative data and Chi-square test/Fishers exact T-test for qualitative variables. P value <0.05 was considered significant.

**Results:**

CD34 was positive in 66.66% (50/75) cases while it was negative in rest (33.33%; 25/75) cases. CD13, CD33, CD5, CD7 and CD11b were more frequently expressed in CD34 negative B-ALL (p=0.025). No association of *CD34* expression was found with gender, age, TLC, FAB morphological type, other immune-phenotypic markers, MRD and cytogenetics studied.

**Conclusions:**

The expression of *CD34* does not associate with known prognostic markers in B-ALL. However, absence of CD34 is associated with aberrant immunophenotypic expression (myeloid + T-cell antigens) in these patients. Larger studies with larger sample size and more extensive immunophenotypic panel needs to be done in Indian setup to confirm these findings.

## Introduction

Acute lymphocytic leukemia (ALL) is the most common hematological malignancy in Indian children. With recent technological advances in the last decade, flow cytometric immunophenotyping on blood or bone marrow sample has become the most widely used technique for lineage determination in leukemias and establishing the diagnosis of B ALL. In addition, it is also used for MRD analysis which is considered the most important prognostic factor following chemotherapy. CD34, a marker of immaturity, is of paramount importance in this regard as it is used both in diagnosis as well as its pattern of expression helps to differentiate blasts from hematogones in a regenerative bone marrow (BM) later.

CD34 is a transmembrane protein that was first identified on hematopoietic stem and progenitor cells. It promotes attachment of these progenitor cells to the components of stromal microenvironment thus promoting their growth and differentiation and mediates resistance to apoptosis. It is a marker of immaturity or blast population in B- acute lymphoblastic leukemia (B-ALL) which usually also express *CD10* (co-expressed in approximately 70% of common B-ALL) and lack CD20 (Basso et al.,2001). As the blasts mature, CD34 and CD10 antigens sequentially decrease in intensity and become negative while the intensity of *CD20* expression on the surface increases (Basso et al.,2001). However, a large percentage (50%) of the phenotypically more immature B blasts that is the pre pre-BALL blasts are negative for both CD10 and CD34, thus accounting for those cases where *CD34* expression is absent (Basso et al.,2001). Yet, CD34 is universally used as one of the markers for blast identification in ALL panel. In the absence of an extensive panel with more markers which may not be available everywhere, there is a risk of missing the diagnosis with such an approach. Further, some studies have also assigned prognostic significance to CD34 as it is reported that the expression of *CD34* is associated with good prognosis in acute myeloid leukemia (Basso et al., 2001). However, literature is sparse on the prognostic significance of *CD34 *expression in B-ALL especially from India. Hence the present study was undertaken to analyse the frequency of *CD34* expression in B-ALL in Indian patients and determine its prognostic significance by associating with other prognostic markers and aberrant antigen expression.

## Materials and Methods

This is a retrospective analysis of data carried out in the Hematology section of Department of Pathology of a tertiary care hospital in Delhi, India over a period of three and a half years. Seventy-five cases diagnosed as B-ALL over these years were evaluated. Complete clinical history, physical examination, and investigations along with immunophenotypic details of these cases were noted from the records.

Complete blood cell count was done on Beckman coulter LH 500. Peripheral blood (PB) and bone marrow aspirate (BMA) smears were stained with Wright’s stain.

Immunophenotye (IPT): IPT of blasts was done from lysed whole peripheral blood (PB) or bone marrow aspirate (BMA) (lyse and wash protocol) using flow cytometer (Beckman coulter cytomics FC 500) equipped with facility for at least 5color IPT. 

The panel used for characterisation of blasts was as follows: Immaturity markers: CD45/CD34/TdT/HLADR, myeloid markers: cMPO/CD117/CD13/CD33/CD64/CD11b, T lymphoid markers: cCD3/CD3/CD5/CD7, B-lymphoid markers: CD79a/CD10/CD19/CD20.

A threshold of 20% was used to define a positive reaction of blast cells to a given monoclonal antibody, except for cMPO, cCD3, cCD79a and TdT, which were considered positive at 10% level of expression (Chiaretti et al., 2014). Patients were diagnosed as B-ALL on the basis of WHO criteria i.e. strong CD19 plus strong expression of at least one of CD79a, cCD22, CD10 or weak CD19 plus strong expression of at least two of *CD79a, cCD22*, *CD10* (Swerdlow et al., 2008).

Cytogenetics analysis: This was done by fluorescence in situ hybridisation (FISH) for t(9;22), t(12;21), t(4;11) at the time of diagnosis. Data on cytogenetics was available for 24/75 cases. 

Minimal residual disease (MRD): Cell preparation was done using stain-lyse-wash method on BMA sample using flow cytometer (Beckman coulter cytomics FC 500) equipped with facility for at least 5color flow panel. Following markers were used: CD45, CD10, CD19, CD20, CD34, CD38, CD58, CD128, CD123, CD86 and CD200. A cut-off of 0.01% was used to define MRD positivity. Data on MRD was available for 25/75 cases.

Statistical analysis was performed using MS EXCEL and SPSS software version 20. Numerical data were expressed as mean and standard error of mean. Qualitative data was expressed as frequency and percentage. Differences between groups were evaluated using Student’s T-test for quantitative data and Chi-square test/Fishers exact T-test for qualitative variables. P value less than 0.05 was considered significant.

## Results

*Clinico-hematological profile*

Out of 75 cases of B-ALL diagnosed, 40 were males and 30 were females with a male to female ratio of 1.3. There were 58 cases in the age group of 2-10 years with a mean±SD age of 4.75±1.9 years and 17 cases in the age group of >10 years with a mean±SD age of 22.56±11.4 years. Total leucocyte count ranged from 1.3 to 580 ×10^9^/L with a median of 25 ×10^9^/L. The mean blast count was 56.6.

*Morphological (French-American-British [FAB]) subtype*

41.4 % (31/75) cases had FAB L1 morphology with presence of regular round homogenous blasts with only little clefting while 58.6% (44/75) cases had FAB L2 morphology with presence of heterogeneous blasts with moderate amount of cytoplasm, vacuoles, prominent nucleoli and prominent indentations and clefting. No case had L3 morphology.

*Immunophenotypic profile*

*CD34 *expression: CD34 was seen in blasts in 66.66% (50/75) cases. Figure 2 shows flow cytometry of a case of CD34 negative B-ALL. HLA DR proved to be more useful for defining immaturity as it was expressed by blasts in 92% (69/75) cases. 

**Figure 1:**
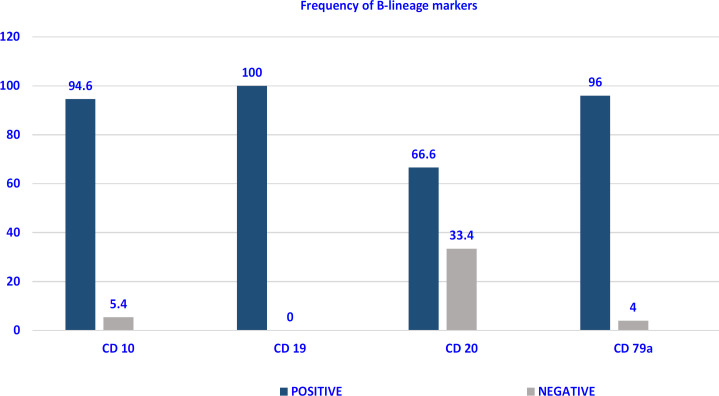
Frequency of B-lineage markers in B-ALL (%) (N=75)

Expression of B lineage markers: CD19 was expressed in all the 75 cases. The next most common B lineage antigen was cytoplasmic CD79a which was expressed in 96% followed by CD10 in 94.6% (Figure 1). Though blasts are immature cells, 66.6% showed a variable expression of *CD20* with asynchronous co-expression of *CD34 *and *CD20* seen in 44% (33/75). So, according to the EGIL classification, there were 4 (5.4%) cases of Pro-B-ALL or EGIL B-1 subtype [(A positive reaction for any two of CD19, CD22 (membrane and cytoplasm) and CD79a) without further differentiation markers] and 71 (94.6%) cases of CD10 positive B-ALL (EGIL B-2) subtype (Chiaretti et al., 2014). 

Aberrant marker expression: A total of 34.6% (26/75) cases exhibited aberrant expression of immunophenotypic markers. The most common aberrant marker expressed was CD33 seen in 14.6% (11/75) cases, followed by CD7 and CD13 seen in 8% (6/75) cases each. CD5 was expressed in 4% (3/75) cases, and CD11b in 1.33% (1/75) cases.

Cytogenetics: Out of the 24 cases where data on cytogenetics was available, 3/24 had a positive cytogenetic analysis at diagnosis. These were positive for t(9;22), t(12;21), and one was a known case of neurofibromatosis 1 (NF1). 

Minimal residual disease: Data on MRD was available for 25/75 cases. Out of these 25, MRD was positive in 5/25, 20% of cases. Out of these 5, 2 had positive cytogenetics at diagnosis for NF1 and t(9;22). 

Comparison between CD34 positive and negative group: The two groups were then compared with respect to aberrant marker expression and prognostic factors (Tables 1 and 2).

With respect to expression of one or more aberrant antigens, the CD34 negative group showed a significantly higher expression of one or more of the following *- CD13, CD33, CD5, CD7*, and *CD11b* (p=0.25) (Table 1).

Comparison of *CD34* expression and other prognostic parameters (Age, TLC, and MRD and Cytogenetics) (Table 2): The CD34 positive and negative group did not show any significant difference with respect to age, cytogenetics and FAB morphology. Though MRD and Cytogenetics were done only in limited number of cases, it was seen that CD34 negativity was not associated with adverse prognosis with respect to MRD or cytogenetics. 

**Table 1:** Immunophenotypic Parameters According to *CD34* Expression in B-ALL (N=75)

Parameter	CD 34 Positive (N=50,%)	CD 34 Negative (N=25,%)	p-value
CD 10			
Negative	02 (4)	02 (8)	0.407
Positive	48 (96)	23 (92)	
CD 20			
Negative	17 (34)	08 (32)	0.538
Positive	33 (66)	17 (68)	
HLADR			
Negative	04 (8)	02 (8)	0.685
Positive	46 (92)	23 (92)	
CD 13 + CD 33			
Negative	42 (84)	16 (64)	0.051
Positive	08 (16)	09 (36)	
CD 13 + CD 33 + CD5 + CD 7 + CD 11b			
Negative	37 (74)	12 (48)	.025*
Positive	13 (26)	13 (52)	

*p <0.05 is statistically significant

**Table 2:** Clinical and Laboratory Parameters According to *CD34* Expression in B-ALL (N=75)

Parameter	CD 34 Positive (N=50,%)	CD 34 Negative (N=25,%)	p-value
Age			
2-10 years	39 (78)	19 (76)	0.531
>10 years	11 (22)	06 (24)	
Sex			
Male	27 (54)	16 (64)	0.283
Female	23 (46)	09 (36)	
Total leucocyte count/mm^3^			
<50,000	33 (66)	18 (72)	0.401
>50,000	17 (34)	07 (28)	
FAB type			
L1	20 (40)	11 (44)	0.465
L2	30 (60)	14 (56)	
MRD (N=25)			
Negative	14 (73.6)	6 (100)	0.219
Positive	5 (26.3)	0 (0)	
Cytogenetics (N=24)			
Negative	16 (84.2)	5 (100)	0.479
Positive	3 (15.8)	0 (0)	

FAB, French-American-British; MRD, Minimal residual disease

**Table 3:** Comparison of Aberrant Antigen Expression in B-ALL in Indian Literature

Serial number	CD33 (%)	CD13 (%)	CD7 (%)	CD5 (%)	CD11b (%)
1. Gujral et al (2009)	6.07	2.78	0.9	NA	NA
2. Birva et al (2019)	14.0	15.0	0.0	0	NA
3. Gupta et al (2019)	17.9	25.6	0.03	NA	NA
4. Sharma et al (2016)	28.1	33.3	2.0	NA	NA
5. Present study (2020)	14.6	8.0	8.0	4	1.33

NA, Not available

**Table 4:** Comparison of Aberrant Myeloid Antigen Expression and *CD34* Expression in ALL in Literature

Serial number	Ethnicity	Age group (years)	Number of patients	MyAg studied	Correlation between MyAg and CD34	p-value*
1. Mi et al (1999)	Chinese	Adult	102	NA	Not found in ALL and B-ALL	NA
2. Yeneral et al (2002)	Turkish	Adult (14-62)	46	CD13, CD33	Not found in ALL	0.06
3. Vitale et al (2007)	European (Rome)	Adult (14-60)	377	CD13, CD33	Present between MyAg+ and CD34+ in ALL	<0.0001*
4. Tong et al (2010)	Chinese	All (1-72)	113	CD13, CD33, CD15, CD117	Present between MyAg+ and CD34+ in ALL	0.002*
5. Tong et al (2011)	Chinese	Pediatric (0-14)	207	CD13, CD33, CD15, CD117	Present between MyAg+ and CD34+ in ALL	0.004*
6. Tong et al (2014)	Chinese	Adult (15-77)	110	CD13, CD33, CD15, CD117	Present between MyAg+ and CD34+ in ALL	0.019*
7. Sharma et al (2014)	Indian	All (2-61)	ALL (204) B-ALL (163)	CD13, CD33, CD117	Present between MyAg+ and CD34high in ALL and B-ALL	0.0001* (in both ALL and B-ALL)
8. Sharma et al (2016)	Indian	All (<1-65)	Adult (96), Pediatric (207)	CD13, CD33, CD117	Present between MyAg+ and CD34+ in ALL	p=0.001* (Adult), p=0.0004* (Pediatric)
9. Present study (2020)	Indian	All	75	CD13, CD33	Not found in B-ALL	0.051

NA, Not available; My, Myeloid antigen expression; My+, Myeloid antigen positivity; ALL, Acute lymphoblastic leukemia; B-ALL, B- acute lymphoblastic leukemia; *p<0.05 is statistically significant

**Figure 2:**
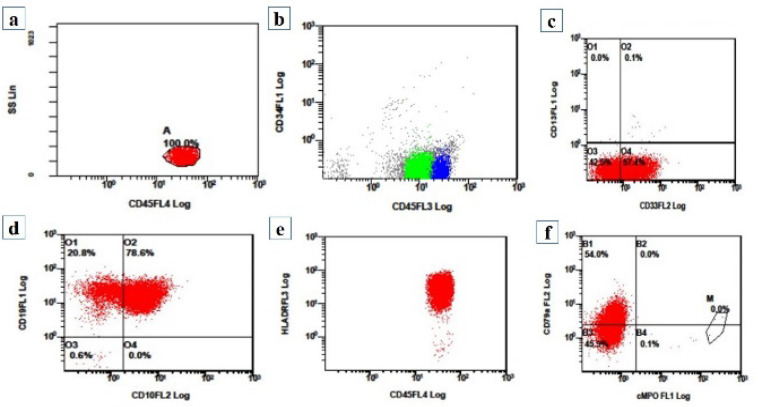
(a-f): Flow cytometry graphs of a case of CD34 negative B-ALL: (a) Side scatter on y-axis and CD45 on x-axis shows a population of dim CD45 positive blasts. (b) CD 34 on y-axis and CD45 on x-axis shows CD34 negative blasts. (c) CD13 on y-axis and CD33 on x-axis shows blast population heterogeneous positive for CD33 while negative for CD13. (d) CD19 on y-axis and CD10 on x-axis show all blasts expressing CD19 while being heterogeneous positive for CD10. (e) HLA-DR on y-axis and CD45 on x-axis shows HLADR positive blasts. (f) CD79a on y-axis and cMPO on x-axis shows CD79a positive blasts while negative for myeloid marker cMPO

## Discussion

The diagnosis and classification of acute leukemia is currently a multistep procedure based on morphology, immunophenotype, cytogenetics and molecular genetics. Flow cytometry immunophenotyping is extensively used in the diagnosis of almost all hematolymphoid neoplasms because of its capacity to analyse multiple markers simultaneously on the same cells and rapid results.

Diagnosis of B ALL is based on lineage assignment (A strong CD19 plus strong expression of at least one of *CD79a, cCD22, CD10* or weak* CD19* plus strong expression of at least two of CD79a, cCD22, CD10) and expression of immaturity markers by the atypical cell population (Chiaretti et al., 2014). CD34, an immaturity marker, usually expressed in leukemia cells is universally used as it unequivocally establishes the immature nature of these cells. However, several studies from the West and some Indian studies have documented that CD34 may not be uniformly expressed in all cases of B ALL. Literature is sparse on the expression of *CD34* expression in B-ALL from India, as well as its prognostic significance is not well explored. Hence the present study was undertaken to analyse the frequency of *CD34* expression and its association with other prognostic markers in B-ALL patients from a tertiary care centre in India.

The frequency of CD34 positivity in B-ALL was 66.6% while 33.3% did not express CD34. The reported frequency of CD34 in B-ALL from previous Indian studies ranges from 30%-81.3% (Gujral et al., 2009; Sharma et al., 2016; Birva et al., 2019; Gupta et al., 2019) and in studies from other parts of the world is also in similar range of 47.8% to 83% (Thalhammer-Scherrer at al., 2002; Dakka et al., 2009; Jaafar et al., 2018; Rezaei et al., 2020). As regards immaturity we found HLA-DR better for delineation of blasts as its expression was seen in 92% cases. The reported frequency of HLA-DR in B-ALL from previous Indian studies is also higher than *CD34 *expression in B-ALL ranging from 97.4%-100% indicating that it’s a better marker of immaturity (Gujral et al., 2009; Birva et al., 2019; Gupta et al., 2019). 

There were 5.7% cases of pro-B-ALL and 94.2% cases of CD10 positive B-ALL. *CD19* expression in 100% of cases and CD79a was positive in 96% cases. Our results are similar to Indian study by Gujral et al., (2009) who found 96% CALLA positive B-ALL, 4% pro-B-ALL, *CD19* expression in 100% B-ALL cases. However, the expression of *CD79a* (99.4%) was higher than the present study. 

Asynchronous co-expression of *CD34* and *CD20* was seen in 44% (33/75) (Table 1) cases. Seegmiller (2009) also reported such an asynchronous expression in 37.5% (75/200) cases of B—ALL cases while Sharma (2016) and Jalal (2017) an asynchronous dual expression of *CD34 *and *CD20* in 12% cases. Firstly, such asynchronous expression of early and late antigens defies normal antigenic evolution of B- cell precursors and thus can be useful to differentiate blasts from hematogones in evaluating MRD. Secondly, *CD20* expression is associated with poor prognosis in B-ALL (Thomas et al., 2009). However, introduction of anti-CD20 monoclonal antibody like rituximab to the traditional chemotherapy regimens can improve the survival outcomes in precursor B-ALL patients (Thomas et al., 2009). 

There was a predominance of L2 morphology than L1 morphology in B-ALL blasts in the present study. Our findings are in contrast to previous studies who found L1 to be more common than L2 morphology in B-ALL (Bennett et al., 1981). 

Aberrant expression is defined as presence of an antigen belonging to some other lineage. This is a well-known phenomenon in leukemias and helps in identification of the malignant cells/blasts. Further, some studies have documented that aberrant expression of myeloid antigens (*CD13* and *CD33*) is associated with better prognosis while that of T-cell antigens (CD5 and CD7) is associated with worse prognosis in B-ALL (Hussein et al., 2011; Ibrahim et al., 2017). In the present study, out of the total 75 cases, 26 showed expression of aberrant markers. The most common aberrant marker expressed in the present study was CD33 seen in 14.6% cases, followed by CD7 and CD13 each seen in 8% cases. Most of the Indian studies have found CD13 to be the most common aberrant marker expressed in B ALL followed by CD33 (Table 3). Table 3 shows the aberrant expression of various markers in B-ALL in Indian literature. 

In this study, a significant association was seen between CD 34 negative B-ALL and expression of one or more aberrant immunophenotypic markers (CD13 + CD 33 + CD5 + CD7 + CD11b) (p=0.025) (Table 1). In a study by Jung (1996), there was a strong correlation between CD 34 negative ALL and aberrant expression of *CD7* and *CD5* only but not myeloid markers. (p=0.0005, p=0.034 respectively). Further, we found absence of *CD34* expression to be associated with a more frequent expression of myeloid markers (*CD13 + CD33*) though the results were not statistically significant (p=0.051, Table 1). This finding is similar to that found by Mi (1999) and Yeneral (2002) who did not find any relationship between myeloid antigen expression and *CD34* positivity in both ALL, subgroup B-ALL and only ALL patients respectively (Table 4). However, other studies found a correlation (Table 4) (Vitale et al., 2007; Tong et al., 2010; Tong et al., 2011; Tong et al., 2014; Sharma eta l., 2014; Sharma et al., 2016). These differences may be due to difference in demographics (ethnicity, age group studied), sample size, patient cohort (ALL instead of B-ALL) and myeloid antigens analysed. Table 4 shows the comparative analysis of aberrant myeloid antigen expression and *CD34 *expression in ALL in literature. 

Absence of CD34 has been considered to be an independent adverse prognostic factor in B-ALL (Basso et al., 2011). CD34 positivity in B-ALL has been found to correlate with good prognostic factors like age 1-10 years, low TLC count and expression of *CD10, CD19*, *HLADR* in various studies (Basso et al., 2001; Dakka et al., 2009). Therefore, an attempt was made to correlate CD34 negativity with other prognostic factors. There was no association between CD34 negativity with age, sex, TLC and expression of *CD10, HLADR* (Table 1,2). Such a difference could be because of different sociodemographic profile and biology of B-ALL in Indian patients. Cytogenetics and MRD was available in only one third of the cases. CD34 negativity was not found to associate with bad cytogenetics or MRD positivity in these cases (Table 2). These findings are in contrast to the findings of studies form other countries as CD34+ is reported to be associated with t(12; 21], adults with t(9; 22) and t(4; 11) in B-ALL while CD34- is associated with t(1; 19) (Basso et al., 2001). These differences could also be because of availability of smaller number of patients with a cytogenetic test. As India being a developing nation, not all patients are able to afford these costly cytogenetic analytic tests. 

To conclude, the expression of *CD34* is not associated with other prognostic markers in B-ALL while absence of *CD34* is associated with aberrant immunophenotypic expression of markers (myeloid + T-cell antigens) in B-ALL. This may be due to the different sociodemographic profile and biology of the disease in Indian patients. Larger studies with larger sample size, more extensive immunophenotypic panel, cytogenetics and follow up survival analysis needs to be done in Indian setup to clearly understand the biology of expression of *CD34* in B-ALL and thus paves the way for future research.

## Author Contribution Statement

Literature search, data acquisition, data analysis, manuscript preparation and manuscript editing was done by NG. Concept, design of the study along with manuscript editing and review was done by RG. Manuscript editing and review was done by MK. 
